# Genetic Variations in Sweet Taste Receptor Gene Are Related to Chocolate Powder and Dietary Fiber Intake in Obese Children and Adolescents

**DOI:** 10.3390/jpm8010007

**Published:** 2018-01-29

**Authors:** Marina B. Pioltine, Maria Edna de Melo, Aritânia S. Santos, Alisson D. Machado, Ariana E. Fernandes, Clarissa T. Fujiwara, Cintia Cercato, Marcio C. Mancini

**Affiliations:** 1 Department of Endocrinology, League of Childhood Obesity, Hospital das Clínicas da FMUSP, Sao Paulo 05403-000, Brazil; medna@usp.br (M.E.d.M.); ariana@usp.br (A.E.F.); clarissafujiwara@usp.br (C.T.F.); ccercato@netpoint.com.br (C.C.); mmancini@usp.br (M.C.M.); 2 Laboratory of Carbohydrates and Radioimmunoassay (LIM18) Faculty of Medicine, University of Sao Paulo, Av. Dr. Arnaldo 455, 3 Andar (Sala 3324), Sao Paulo 01246-903, Brazil; 3 Faculdade de Medicina da Universidade de São Paulo, Sao Paulo 01246-903, Brazil; aritania@usp.br (A.S.S.); alissondiegomachado@hotmail.com (A.D.M.)

**Keywords:** TAS1R2, genotype, obesity, food intake, polymorphism

## Abstract

Childhood obesity is a major public health problem. It has a direct impact on the quality of life of children and adolescents, as well as on their future risk of developing chronic diseases. Dietary patterns rich in fats and sugars and lacking dietary fibers, vitamins, and minerals, as well as lack of physical exercise have been associated with the rise of obesity prevalence. However, factors that contribute to the preference for foods rich in these nutrients are not well established. Taste is recognized as an important predictor of food choices, and polymorphisms in taste-related genes may explain the variability of taste preference and food intake. The aim of this research is to evaluate the influence of polymorphisms of the sweet taste receptor gene *TAS1R2* on diet and metabolic profile in obese children and adolescents. A cross-sectional study with 513 obese children and adolescents and 135 normal-weight children was carried out. A molecular study was performed for the single nucleotide polymorphisms (SNPs) rs9701796 and rs35874116 of *TAS1R2*, and dietary intake, anthropometric parameters (weight, height, waist circumference, waist-to-height ratio (WHtR)), and metabolic profile (including fasting glucose, insulin, triglyceride, high-density lipoprotein (HDL)–cholesterol, and leptin levels) were analyzed. The variant rs9701796 was associated with increased waist-height ratio, as well as with a higher chocolate powder intake in obese children. The variant rs35874116 was associated with a lower dietary fiber intake. In conclusion, there was no relationship between genotypes and risk of obesity. Obese adolescents carrying the serine allele of SNP rs9701796 in TAS1R2 showed higher waist-to-height ratio and chocolate powder intake, whereas those carrying the valine allele of SNP rs35874116 in TAS1R2 were characterized by lower dietary fiber intake.

## 1. Introduction

Taste is the sense of the human body that allows the recognition and evaluation of eatable foods. Humans are able to recognize five basic types of taste: sweet, salty, bitter, sour, and umami [1]. Recently, the oral perception of dietary fat, named *Oleogustus*, was described as the sixth type of basic taste [2]. Food choices involve physiological, nutritional, environmental, and sociocultural aspects, but genetics also plays an important role [3]. However, the sensory qualities of food are essential to food preference, being important for adherence to dietary changes [4,5].

The sweet taste receptor is a heterodimer of two protein subunits, T1R2 (taste receptor, type 1, member 2), which is specific for the sweet taste and is composed of 839 amino acids, and T1R3 (taste receptor, type 1, member 3), responsible for the perception of the sweet and umami tastes [6]. Sweet substances include breast milk, added sugars, and sugars naturally present in foods and beverages [7]. The sweet taste has good palatability and innate attraction, and it is well established that the consumption of sweet foods stimulates the reflexes of cephalic phase that are important for the beginning of digestion [6,8]. Furthermore, taste receptors also seem to have an effect on other parts of the digestive system such as the stomach and intestine, leading to afferent responses via vagus nerve, which can modulate the sensibility of taste and participate in new regulatory pathways of energy homeostasis [8,9].

The gene *TAS1R2*, responsible for encoding the T1R2 protein (sweet taste receptor), is located on chromosome 1p36.13 and has six exons [10]. A few studies evaluated the effect of variations in the gene *TAS1R2* on the perception of the sweet taste. Fushan et al. [11] performed DNA sequencing to identify the effect of single nucleotide polymorphisms (SNPs) in *TAS1R2* on the ability of individuals to discriminate sucrose solutions at different concentrations. Dias et al. found that another polymorphism in this gene, rs12033832, is associated with the threshold of perception of the sweet taste and with sugar intake, but its effect changes according to the body mass index (BMI) [12].

Eny et al. evaluated the effect of two missense SNPs in *TAS1R2*, including rs9701796, which leads to a replacement of serine for cysteine at codon 9 (Ser9Cys), and rs35874116, which leads to a replacement of isoleucine for valine at codon 191 (Ile191Val), on sugar intake. There was a significant SNP–BMI interaction for Ile191Val and sugar intake [13]. Among overweight individuals, it was observed that valine carriers consumed significantly lower amounts of sugars than those who were homozygous for isoleucine [13]. Variations in *TAS1R2* also have been associated with the perception of the sweet taste and the risk of dental caries, which is directly related to sugar intake. However, none of these studies evaluated the quantitative sugar intake in children with different BMIs [14,15,16,17].

Considering the importance of understanding potential influencers in food consumption and genetics that can contribute to the development of obesity, the aim of this study was to evaluate the influence of SNPs rs9701796 and rs35874116 in *TAS1R2* in adiposity, dietary intake, and metabolic profile in children and adolescents.

## 2. Methods

We conducted a cross-sectional study with children and adolescents aged 7 to 18 years. Data collection was performed between December 2012 and May 2015. We evaluated 513 obese children and adolescents from the Endocrinology Clinic of the Hospital das Clínicas (São Paulo, Brazil) and 135 normal-weight children from public schools. All parents or guardians provided written informed consent before participation. The University of São Paulo Ethics Committee approved the study (process CAAE 12247613.0.0000.0068). Key exclusion criteria were the use of medications that cause significant weight change, and significant medical chronic conditions or genetic syndromes.

We evaluated weight, height, waist circumference (at the midpoint between lower rib and iliac crist), waist-to-height ratio (WHtR), blood pressure, body composition by bioimpedance analysis (RJL Systems^®^, model Quantum II, Charter Twp of Clinton, MI, USA), and metabolic profile, including fasting blood glucose, lipid profile (total cholesterol, high-density lipoprotein–cholesterol (HDL–c), low-density lipoprotein–cholesterol (LDL–c), triglyceride levels), insulin and leptin levels, and leptin adjusted by weight and Homeostasis Model Assessment for Insulin Resistance (HOMA-IR) calculation [18]. The classification of the BMI z-score (zBMI) was performed by AnthroPlus^®^ [19].

The dietary intake was assessed by two 24 h recalls taken 30 days apart, using the Multiple Pass Method [20,21]. This method contributes to the remembrance of foods and drinks consumed in the day before the interview, reducing biases in the dietary measure. For the analysis of food intake, household measures present in 24 hour-recalls were converted to grams [22]. Total energy, macronutrients (carbohydrates, protein, total fat), fractions of dietary fatty acids including saturated (SFA), monounsaturated (MUFA), and polyunsaturated (PUFA) fats, and dietary fiber values were calculated using the Brazilian Table of Food Composition [23]. Total sugars were calculated according the United States Department of Agriculture (USDA) Food Composition Database [24].

We also evaluated the individual intake of the most cited sweet foods in the food recalls. Foods were rated as sweet when contained added sugar or at least 1 g of fructose for every 100 g of food [24]. We excluded participants who had a caloric consumption lower than 500 kcal or higher than 5000 kcal (*n* = 23) (improbable values that could lead to under- or overestimation of the nutrient intake) [25]. Nutrient intakes were adjusted for energy intake using the residual method [26].

A molecular study was performed in DNA samples extracted from white blood cells, and in normal-weight subjects in DNA extracted from saliva samples (Oragene DNA G-500^®^, DNA Genotek Inc., Ontario, CA, USA) in obese patients. The genotyping of polymorphisms was performed using the TaqMan assay ID c_27269371_20 e c_55646_2 by real-time PCR (Applied Biosystems, Foster City, CA, USA).

The frequencies of polymorphisms in *TAS1R2* were tested using the chi-square test for calculation of Hardy–Weinberg Equilibrium (HWE). The analyses of polymorphisms were conducted by the dominant model, in which homozygotic individuals for the ancestral allele and individuals carrying the polymorphic allele are compared [27]. We used the JMPv.10 software (SAS Institute Inc., Cary, NC, USA) for the comparison of frequencies of polymorphisms. For this, we performed a logistic regression analysis adjusted for confounding variables, and the magnitude of risk was estimated by odds ratio (OR) and its 95% confidence intervals (95% CI). 

Continuous variables were submitted to the Kolmogorov–Smirnov test to verify the normality. We used the Student *t*-test for variables with symmetrical distribution and the Mann–Whitney test for variables with asymmetric distribution. A linear regression analysis was performed between polymorphisms and nutrient intake as a dependent variable. Statistical tests were performed using the SPSS software (SPSS Inc., Chicago, IL, USA), version 22.0. Values were considered statistically significant when *p* < 0.05.

## 3. Results

The study sample consisted of 513 obese children and adolescents, being 52.7% males and 47.3% females. The mean age of the obese children was 12.2 ± 3.1 years old, and the mean zBMI was 3.2 ± 0.7. The normal-weight children (*n* = 135) were 55.5% male and 44.5% female, with a mean age of 10.4 ± 0.9 years old and a mean zBMI of 0.2 ± 0.7. All 513 obese participants had anthropometric, clinical, biochemical, and genetic data collected. Of these, 297 (57.9%) individuals had the dietary intake assessed. All 135 normal-weight children had anthropometric measurements, genetic data, and dietary intake collected.

Table 1 shows the frequency of each genetic variant. None of them was related to the risk of obesity. Obese individuals carrying the valine allele of rs9701796 in TAS1R2 presented higher waist-to-height ratio. There was no association between other parameters analyzed in obese and normal-weight children, and there was no difference in rs35874116 genotypes (Table 2).

Regarding the dietary intake, when the most consumed sweet foods were evaluated, we observed a higher chocolate powder intake in obese children with the allelic variant (15 g—10–20 g— versus 20 g—16–20 g—; *p* = 0.04) (Table 3).

In the descriptive analyses, there was a greater intake of MUFA (g and %) in serine-carriers obese children, as well as a marginal difference in total fat intake (g and %) (Table 4). In the regression analyses, there was no association between the intake of these nutrients and the genetic variants, both in the univariate and in the multivariate analysis, with adjustments for age and sex.

Furthermore, we identified that obese carriers of the valine allele in rs35874116 presented a lower dietary fiber intake, which was not observed in normal-weight children. In the multivariate regression analysis this association remained after adjusting for age and sex (Table 5). 

## 4. Discussion

A few studies have investigated the effect of genetic variations in the sweet taste receptor gene on the perception of taste or on sugar intake [11,12,13]. The present study is the first to identify a variant in a sweet taste receptor gene that affects both sweet food intake and metabolic risk. The obese carriers of the serine allele of rs9701796 in TAS1R2 showed higher metabolic risk according to the waist-to-height ratio, as well as higher chocolate powder intake. Eny et al. evaluated this variant and its relationship with dietary intake and nutritional status, but no associations were found [13], which could be explained by the fact that their study was performed in adults, while the population of our study was composed of children and adolescents.

Also noteworthy is that leptin acts on taste receptors and specifically inhibits the taste responses to the sweet taste without affecting the responses to the sour, salty and bitter tastes, which is not observed in leptin receptor deficiency. This indicates that this hormone can be a modulator of the perception of the sweet taste, with a direct action in the regulation of the dietary intake [28]. The higher chocolate powder intake in obese children and adolescents observed in this study may be related to leptin, which is known to increase the sucrose and glucose taste thresholds. Furthermore, higher leptin levels are associated with higher BMI [29]. In this study, obese children carrying the serine allele of SNP rs9701796 consumed more chocolate powder. However, there was no association with other sweet tasting foods.

We also observed that the rs35874116 variant was associated with a lower dietary fiber intake in obese children, and therefore can be considered a risk variant for this dietary pattern. Due to the small number of participants with these variants and different sugar intakes, it was not possible to establish a conclusion regarding sweet-tasting food intake. This variant has been the subject of study for research on the risk of caries, using as analytical method the presence of decayed teeth. Studies performed with children from the Czech Republic [14] and Turkey [15] showed that the T allele (carriers of valine) was associated with a higher incidence of tooth decay. Although the dietary intake was not analyzed in these studies, it is known that the presence of tooth decay is directly related to sugar intake, independently of other factors [30,31]. However, a study conducted in the Canadian population found that this same variant was associated to a lower presence of tooth decay in adults [16], which could be explained by factors related to oral hygiene, food preference, and a different dietary intake during childhood and adulthood.

Eny et al. also verified that in individuals with overweight and obesity, the SNP rs35874116 in TAS1R2 gene was associated with a lower dietary fiber intake [13], as we found in our study. However, these same participants also presented a lower carbohydrate and sugar intake. This association was not found in our study, possibly because of the age difference of the participants. Age may modify eating habits since the presence of sugar is related to the taste of foods, while this is not necessarily the case for the fibers.

Several factors may be involved in food preference at different stages of life. The preference for sweet taste seems to be innate in humans. Almost all newborns positively react to sugar solutions as opposed to lemon juice solutions, according to their facial expressions [32]. In a study conducted with children (9–10 years old), adolescents (14–16 years old), and adults (20–25 years old) that investigated the perception of intensity of the sweet taste in solutions containing different sugar levels, the authors observed that children had a higher preference for high concentrations of sugar than teenagers, who had a higher preference than adults. Age had a similar effect when water with sucrose and orange juice with sucrose were evaluated [33].

These studies suggest that the sensitivity to taste may change with age. A possible biological explanation could be the higher demand for energy by children. Furthermore, sugar is a perceptible nutrient in food and is related to energy content, while the bitter taste is anthropologically related to toxic or poisonous food that the body is programmed to avoid [34,35,36,37,38].

There was no difference in the dietary intake according to the sex in our population, although boys and girls may also differ in their food intake especially after puberty [39]. Gender differences in taste perception have been reported as well, however we did not evaluate the taste perception thresholds [40].

The present study used 24 hour-food recalls by the multiple-pass method to assess the dietary intake in our population, since it can represent the habitual food intake when doubly applied in typical days. Nevertheless, studies on food intake are estimates and can present limitations when not standardized or comprehensive. The strengths of our study include a large sample size and a thorough and standardized genetic analysis.

Also, it is worthwhile to mention that neuronal factors can have an important impact on food behavior, since genetic variants can be related to the food intake, but not necessarily to the preference for the food consumed [41,42,43]. We highlight the importance of taste perception tests, as well as the development of a food preference questionnaire regarding the sweet taste in future studies, in order to contribute to the understanding of food behavior in obese children and adolescents [44].

In conclusion, the polymorphisms rs9701796 and rs35874116 in *TAS1R2* gene were not associated to an increased risk of obesity. Obese adolescents carrying the serine allele of SNP rs9701796 in TAS1R2 showed a higher waist-to-height ratio and a higher chocolate powder intake. The valine allele in rs35874116 in TAS1R2 was associated with a lower dietary fiber intake in obese children and adolescents.

## Figures and Tables

**Table 1:** Frequency of genotypes and risk of obesity according to SNPs in *TAS1R2*.

SNP	Normal Weight (*n* = 135)	Obese (*n* = 513)	OR * (95% CI)	*p*
rs9701796				0.27
Cys/Cys	62.3%	64.9%	0.77 (0.49–1.22)	
Ser carriers	37.7%	35.1%		
rs35874116				0.92
Ile/Ile	9.8%	7.8%	1.04 (0.49–2.18)	
Val carriers	90.2%	92.2%		

SNP = single nucleotide polymorphism. OR: odds ratio; CI: confidence interval. * Logistic regression adjusted by sex and age.

**Table 2:** Clinical and biochemical data of obese children and adolescents according to the genotypes of rs9701796 and rs35874116.

Variable	rs9701796		*p*	rs35874116		*p*
	Cys/Cys	Ser Carriers		Ile/Ile	Val Carriers	
	(*n* = 333)	(*n* = 180)		(*n* = 40)	(*n* = 473)	
zBMI	3.28 ± 0.73	3.38 ± 0.86	0.54	3.41 ± 0.85	3.3 ± 0.77	0.94
Waist-to-height ratio	0.66 ± 0.08	0.68 ± 0.09	0.018	0.65 ± 0.09	0.66 ± 0.08	0.19
SBP (percentile)	79 (54–91)	82 (59–91)	0.17	87 (68–93)	79 (56–91)	0.18
DBP (percentile)	66 (48–82)	63 (42–81)	0.68	67 (43–80)	64 (46–81)	0.82
Lean mass (%)	60 ± 6	60 ± 7	0.43	62 ± 6	60 ± 6	0.08
Fat mass (%)	39 ± 6	39 ± 7	0.44	37 ± 6	39 ± 6	0.08
Fasting glucose (mg/dL)	80 ± 10	79 ± 9	0.57	80 ± 8	79 ± 10	0.96
Insulin (µU/mL)	21 (14–33)	22 (14–32)	0.52	18 (10–31)	21 (14–32)	0.28
HOMA-IR	4.11 (2.67–6.76)	4.11 (2.73–6.32)	0.95	3.39 (2.00–6.02)	4.13 (2.70–6.62)	0.20
Total cholesterol (mg/dL)	164 (147–186)	167 (148–193)	0.26	159 (143–189)	165 (148–188)	0.25
LDL–c (mg/dL)	101 ± 29	104 ± 31	0.19	95 ± 27	103 ± 30	0.17
HDL–c (mg/dL)	42 (36–49)	43 (38–49)	0.33	44 (35–49)	42 (37–49)	0.96
Triglycerides (mg/dL)	104 (78–139)	100 (75–144)	0.94	104 (64–133)	103 (77–139)	0.56
Leptin (ng/mL)	40 (26–53)	44 (28–59)	0.18	45 (25–61)	42 (27–56)	0.71
Ajusted leptin (ng/mL/Kg)	1.36 ± 0.73	1.40 ± 0.72	0.53	1.59 ± 0.71	1.35 ± 0.71	0.07

Parametric data are presented as means ± standard deviations with *p*-values determined by t test; nonparametric data are presented as medians (25th–75th percentiles) with *p*-values determined by Mann–Whitney U test. SBP: systolic blood pressure; zBMI: body mass index z-score; DBP: diastolic blood pressure; HOMA-IR: Homeostasis Model Assessment for Insulin Resistance; LDL–c: low-density lipoprotein–cholesterol; HDL–c: high-density lipoprotein–cholesterol.

**Table 3:** The most consumed sweet food items among children and adolescents according to genetic variants of *TAS1R2* gene.

Sweet Taste Food	Normal Weight		*p*	Obese		*p*
	rs9701796			rs9701796		
	Cys/Cys	Ser Carriers		Cys/Cys	Ser Carriers	
	(*n* = 84)	(*n* = 51)		(*n* = 178)	(*n* = 119)	
Chocolate powder (g)	10 (10–20)	20 (10–25)	0.19	15 (10–20)	20 (16–20)	0.04
White sugar (g)	10 (10–18)	10 (10–20)	0.11	10 (10–20)	10 (10–20)	0.56
Soft drinks (mL)	300 (200–400)	250 (187–400)	0.70	350 (200–600)	300 (200–500)	0.41
	rs35874116			rs35874116		
	Ile/Ile	Val Carriers		Ile/Ile	Val Carriers	
	(*n* = 12)	(*n* = 123)		(*n* = 23)	(*n* = 274)	
Chocolate powder (g)	10 (10–10)	18 (10–25)	0.16	10 (10–10)	20 (10–20)	0.12
White sugar (g)	20 (15–30)	10 (10–20)	0.19	20 (10–25)	10 (10–20)	0.20
Soft drinks (mL)	400 (290–500)	300 (200–400)	0.53	350 (200–538)	300 (200–600)	0.90

Nonparametric data are presented as medians (25th–75th percentiles) with *p* values determined by Mann–Whitney *U*-test.

**Table 4:** Dietary intake according to rs9701796 in *TAS1R2*.

Variable	Normal Weight		*p*	Obese		*p*
	Cys/Cys	Ser Carriers		Cys/Cys	Ser Carriers	
	(*n* = 84)	(*n* = 51)		(*n* = 178)	(*n* = 119)	
Energy (kcal/day)	1620 ± 594	1727 ± 784	0.39	1820 (1452–2322)	1775 (1368–2380)	0.48
Carbohydrates (%)	50.2 ± 8.3	50.8 ± 10.0	0.72	56.4 ± 8.8	54.2 ± 9.4	0.39
Carbohydrates (g/day)	199 ± 32	201 ± 38	0.68	257 ± 40	246 ± 43	0.39
Protein (%)	18.5 ± 6.1	18.3 ± 6.0	0.88	15.9 (13.5–19.4)	16.0 (13.5–18.2)	0.51
Protein (g/day)	73 ± 24	73 ± 24	0.92	72 (61–88)	73 (61–83)	0.51
Total fat (%)	31.3 ± 7.2	30.9 ± 8.9	0.77	26.8 ± 7.1	29.4 ± 8.0	0.053
Total fat (g/day)	55 ± 13	55 ± 17	0.90	54 ± 14	59 ± 16	0.054
SFA (%)	13.5 ± 3.4	13.5 ± 4.2	0.93	11.1 ± 3.4	12.2 ± 3.8	0.11
SFA (g/day)	23.8 ± 5.9	23.9 ± 8.1	0.93	22.5 ± 6.9	24.6 ± 7.7	0.11
PUFA (%)	11.6 (9.7–13.7)	10.8 (9.2–12.7)	0.60	4.8 (3.6–6.7)	5.3 (3.6–7.2)	0.32
PUFA (g/day)	20.4 (17.1–23.9)	19.0 (16.2–22.5)	0.57	9.8 (7.2–13.5)	10.6 (7.3–14.7)	0.33
MUFA (%)	5.3 ± 2.2	5.3 ± 2.4	0.99	9.8 ± 2.9	10.9 ±3.6	0.04 *
MUFA (g/day)	9.4 ± 4.0	9.4 ± 4.2	1.00	19.7 ± 5.9	21.9 ± 7.3	0.04 *
Dietary fiber (g/day)	10.7 ± 3.6	11.9 ± 5.1	0.16	14.8 ± 6.0	14.9 ± 6.2	0.63
Total sugars (g/day)	47 ± 20	49 ± 31	0.64	51 (37–75)	52 (34–68)	0.62

Adjusted for energy by the residual method. Parametric data are presented as means ± standard deviations with *p* values determined by *t* test; nonparametric data are presented as medians (25th–75th percentiles) with *p* values determined by Mann–Whitney *U* test. SFA: saturated fatty acid; PUFA: polyunsaturated fatty acid; MUFA: monounsaturated fatty acid. * Univariate: β: 1.54, 95% CI −0.37, 3.45, *p* = 0.11. Multivariate: β: 1.46, 95% CI −0.46, 3.38. *p* = 0.14. Adjusted for age and sex.

**Table 5:** Dietary intake according to rs35874116 in TAS1R2.

Variable	Normal Weight		*p*	Obese		*p*
	Ile/Ile	Val Carriers		Ile/Ile	Val Carriers	
	(*n* = 12)	(*n* = 123)		(*n* = 23)	(*n* = 274)	
Energy (kcal/day)	1650 ± 591	1647 ±688	0.21	2150 (1479–2631)	1769 (1407–2339)	0.20
Carbohydrates (%)	51.4 ± 9.9	50.5 ± 8.9	0.70	56.9 ± 8.0	54.5 ± 9.0	0.28
Carbohydrates (g/day)	201 ± 35	200 ± 35	0.70	258 ± 36	248 ± 41	0.29
Protein (%)	17.4 ±4.4	18.4 ± 6.2	0.21	15.0 (13.5–17.1)	16.1 (13.5–19.9)	0.52
Protein (g/day)	68 ± 18	73 ± 25	0.19	68 (61–78)	73 (61–91)	0.53
Total fat (%)	31.2 ±7.6	31.1 ±7.9	0.60	26.6 ± 6.7	28.3 ± 7.6	0.35
Total fat (g/day)	55 ±14	55 ± 15	0.72	54 ± 13	57 ± 15	0.34
SFA (%)	12.3 ± 3.0	13.5 ± 3.8	0.77	11.1 ± 3.4	11.7 ± 3.6	0.44
SFA (g/day)	21.6 ± 5.3	24.0 ± 6.9	0.65	22.4 ± 6.9	23.7 ± 7.2	0.44
PUFA (%)	10.7 (9.6–12.7)	11.3 (9.4–13.3)	0.59	9.4 ± 2.4	10.4 ± 3.2	0.19
PUFA (g/day)	18.8 (16.4–23.0)	20.1 (16.5–23.1)	0.55	18.9 ± 4.9	21.0 ± 6.6	0.19
MUFA (%)	6.4 ± 3.1	5.3 ± 2.3	0.50	3.8 (2.6–6.1)	5.0 (3.6–7.1)	0.34
MUFA (g/day)	11.2 ± 5.7	9.3 ± 4.0	0.52	7.6 (5.2–12.4)	10.1 (7.2–14.3)	0.34
Dietary fiber (g/day)	12.0 ± 5.9	11.0 ± 4.1	0.36	18.7 ± 9.4	14.3 ± 5.5	0.002 *
Total sugars (g/day)	42 ± 18	48 ± 25	0.86	58 (39–66)	51 (35–74)	0.86

Note: Adjusted for energy by the residual method. Parametric data are presented as means ± standard deviations with *p* values determined by *t* test; nonparametric data are presented as medians (25th–75th percentiles) with *p* values determined by Mann–Whitney *U* test. SFA, saturated fatty acid; PUFA, polyunsaturated fatty acid; MUFA, monounsaturated fatty acid. * Univariate: β: −4.34. 95% CI −7.13, −1.55. *p* = 0.002. Multivariate: β: −4.24. 95% CI −7.01, −1.46. *p* = 0.003. Adjusted for age and sex.

## Abbreviations

TAS1R2	taste receptor, type 1, member 2, gene
zBMI	body mass index, z score
PCR	polymerase chain reaction
SNP	single nucleotide polymorphism
HDL–c	high-density lipoprotein, cholesterol
LDL–c	low-density lipoprotein, cholesterol
SFA	saturated fatty acid
MUFA	monunsaturated fatty acid
PUFA	polyunsaturated fatty acid
